# Transcriptional Regulation and Biological Functions of Selenium-Binding Protein 1 in Colorectal Cancer In Vitro and in Nude Mouse Xenografts

**DOI:** 10.1371/journal.pone.0007774

**Published:** 2009-11-16

**Authors:** Nicole M. Pohl, Chang Tong, Wenfeng Fang, Xiuli Bi, Tianhong Li, Wancai Yang

**Affiliations:** 1 Department of Pathology, University of Illinois at Chicago, Chicago, Illinois, United States of America; 2 The Open Laboratory for Overseas Scientists, Wuhan University College of Medicine, Wuhan, China; 3 Department of Oncology, Zhongnan Hospital, Wuhan University, Wuhan, China; 4 Division of Hematology and Oncology, University of California Davis Cancer Center, Sacramento, California, United States of America; 5 University of Illinois at Chicago Cancer Center, Chicago, Illinois, United States of America; University of Barcelona, Spain

## Abstract

**Background:**

It has been shown that selenium-binding protein 1 (SBP1) is significantly downregulated in different human cancers. Its regulation and function have not yet been established.

**Methodology and Principal Findings:**

We show that the SBP1 promoter is hypermethylated in colon cancer tissues and human colon cancer cells. Treatment with 5′-Aza-2′-deoxycytidine leads to demethylation of the SBP1 promoter and to an increase of SBP1 promoter activity, rescues SBP1 mRNA and protein expression in human colon cancer cells. Additionally, overexpression of SBP1 sensitizes colon cancer cells to H_2_O_2_-induced apoptosis, inhibits cancer cell migration in vitro and inhibits tumor growth in nude mice.

**Conclusion and Significance:**

These data demonstrate that SBP1 has tumor suppressor functions that are inhibited in colorectal cancer through epigenetic silencing.

## Introduction

Selenium binding protein 1 (SBP1) is a 56-KDa protein which is expressed in various cell types, including the heart, liver, kidney, lung and intestine. Human SBP1 was first cloned in 1997 [1] and has been suggested to mediate the intracellular transport of selenium [2]. SBP1 has also been proposed to serve as a marker in colonic cell differentiation and recently, SBP1 has been shown to be a target of the hypoxia-inducible factor-1 alpha (HIF1α) [3] and to directly interact with von Hippel-Lindau protein (pVHL) which may play a role in the proteasomal degradation pathway in a selenium dependent manner [4]. SBP1 has been shown to be decreased in various epithelial cancers, including prostate [5], stomach [6], ovaries [7], lungs [8] and colorectal cancers [9], [10]. Furthermore, low expression levels of SBP1 in colorectal cancer were associated with poor prognosis [9], [10]. Similar results have been observed in lung adenocarcinomas [8] and pleural mesotheliomas [11]. Based on these studies it has been proposed that SBP1 may play a critical role in regulating cancer growth and progression. However, the role of SBP1 in these pathways has not been elucidated.

Epigenetic alterations cause gene silencing, leading to loss of gene expression and function. Two commonly mechanisms have been observed: histone modification and DNA methylation. The latter occurs at cytosine residues in cytosine-guanine (CpG) sequences. Methylation of CpG islands at the promoter is often an early event in tumor progression and is a common mechanism of gene silencing in cancers, occurring in more than 60% of tumor suppressors [12]–[14].

SBP1 has been identified to have 2 CpG islands in its 5′-untranslated region, one of which being close enough to have an impact on the SBP1 promoter regulation (−402 to −613). It is therefore possible that the SBP1 promoter, in tumors with low expression levels of SBP1, may be methylated. Indeed, our data reveal that the SBP1 promoter is hypermethylated in human colon cancer tissues and in human colon cancer cell line HCT116, but a general demethylating agent 5′-Aza-2′-Deoxycytidine (or 5′-aza-decitabine, DAC) restores SBP1 mRNA and protein expression by demethylating the SBP1 promoter region and increasing SBP1 promoter activity. We furthermore provide the first evidence to show that SBP1 has anti-cancer functions - overexpression of SBP1 in HCT116 cells induces H_2_O_2_ -mediated apoptosis, inhibits cell migration *in vitro* and inhibits tumor growth in nude mice.

## Materials and Methods

### Cell Culture, Human Colon Cancer Tissues and Reagents

Human colon cancer cell line HCT116 was cultured in McCoy's 5A media supplemented with 10% FBS, 1% anitbiotic-antimyocytic (GIBCO) and maintained at 37°C in a humidified incubator with 5% CO_2_. Human colon cancer cell lines LS174T, Caco2, HT29 and SW480 were maintained in Minimal Essential Medium (MEM) and maintained under the same conditions as HCT116 cells. Human colon cancer tissues and matched normal tissues have been used by our laboratory recently [10]. For H_2_O_2_ treatment, cells at 80% confluence were treated with 0.3 mM H_2_O_2_ for 24 hours. For demethylation studies, cells were treated with 10 and 30 µM of 5′-Aza-2′-Deoxycytidine (DAC) for 72 or 96 h (Sigma, St. Louis, MO).

### Plasmids Construction

Human SBP1 cDNA was amplified and subcloned into pIRES2 vectors (Clontech, Mountain View, CA) (pIRES2-SBP1) using XhoI and BamHI restriction enzyme sites. The following primers were used: forward: 5′-ATCTCGAGCTATGGCTACGAAATGTGGGAAT-3′ and reverse: 5′-ATGGATCCTCAAATCCAGATGTCAGAGCTAC-3′. To generate HA-SBP1 plasmid, human SBP1 cDNA was amplified and subcloned into HA-tagged pcDNA3.1 vector (pHA) (Invitrogen, Carlsbad, CA) using XbaI and XhoI restriction sites. The following primers were used: forward: 5′-ATCTCGAGATGGCTAC GAAATGTGGGAATT-3′ and reverse: 5′-ATTCTAGATCAAATCCA GATGTCAGAGCTAC-3′. For luciferase assays full length of the SBP1 promoter (−2572 to +1) (pGL4-SBP1) was cloned into the pGL4 vector (Promega Corporation, Madison, WI) using XhoI and HindIII restriction sites. The following primers were used: forward 5′-ATCTCGAGCCCATACATACCA AGCAA -3′ and reverse 5′-TCAAGCTTCGGGTTTGCTGTGCTGGTGTC-3. Accuracy of all plasmids was confirmed via sequencing.

### Transfection

Transient transfection of HCT116 cells with pHA-SBP1 or pHA vector control was performed via Lipofectamine 2000 (Invitrogen, Carlsbad, CA). 24–48 h after transfection, cells were subject to different assays. For stable transfection, HCT116 cells were transfected with pIRES2-SBP1 or pIRES2 (empty vector) alone via Lipofectamine 2000 (Invitrogen, Carlsbad, CA). Stably transfected cells were selected with G418.

### Immunoblotting

For immunoblotting, cells were collected 72 hours after treatment. Cells were lysed using 1xRIPA buffer (Upstate Biotechnology, Lake Placid, NY) containing a protease inhibitor cocktail (Sigma, St. Louis, MO). After cell lysis, 30 µg of protein was loaded on a 10% SDS gel followed by transfer to PVDF membrane. Monoclonal antibody against SBP1 was purchased from MBL.

International Corporation (Watertown, MA; 1∶1000), the antibody against β-actin was purchased from (Sigma, St. Louis, MO; 1∶10000). Secondary anti-mouse antibody was purchased from Santa Cruz Biotechnology (Santa Cruz, CA; 1∶3000). The detected signals were visualized by an enhanced chemiluminescence reaction system, as recommended by the manufacturer (ECL-Plus, Amersham, Piscataway, NJ).

### Methylation-Specific PCR (MS-PCR)

HCT116 cells were treated with or without DAC for 96 hours followed by DNA purification (DNeasy, Qiagen, Valencia, CA). Purified DNA was then converted using the EZ-DNA bisulifide conversion kit from Zymo Research (Orange, CA), which converts cytosine residues to uracil, but does not affect 5-methylcytosine, thus allowing one to identify methylated sequences in the DNA by subsequent MS-PCR. For MS-PCR, methylation and unmethylation specific primers (designed by methylation identification software) [15] complementary to the CpG island spanning −463 to −673 of the 5′ untranslated region were used. PCR was then performed using equal amounts of DNA and the iTaq^TM^ DNA ploymerase (Bio-Rad, Hercules, CA) under the following conditions: initial polymerase activation at 95°C for 5 minutes, 95°C for 45 sec, 53°C (methylated product) or 50°C (unmethylated product) for 45 sec followed by elongation at 72°C for 45 minutes. After 35 cycles, the reaction was furthermore incubated at 72°C for 10 minutes. The total PCR product was then run on a 2% agarose gel. Accuracy of product was determined via sequencing.

### Luciferase Assay

HCT116 cells were transfected with a pGL4 empty vector or pGL4-SBP1 plasmid which contains the full length SBP1 promoter. Renilla was co-transfected as an internal control. 24 h after transfection, cells were treated with PBS or 5′-Aza-2′-Deoxycytidine for 72 h. The dual luciferase activity kit (Promega, Madison, WI) was used in combination with a luminometer to measure luciferase activity of treated cells according to the manufacturers' protocol.

### Proliferation, Apoptosis and Migration Analysis

Cell proliferation was analyzed by MTS assay according to the manufacture's protocol (Promega, Madison, WI). To detect apoptosis, stably transfected HCT116-SBP1 or vector control cells were harvested and fixed with 70% ethanol followed by propidium iodide staining. Cells were then counted by flow cytometry (FACScan, BD Biosciences, San Jose, CA). Cell migration was detected using a transwell plate (Corning, Lowell, MA) followed by DAPI staining.

### Xenografts

1×10^6^ HCT116 cells stably expressing pIRES2-SBP1 or pIRES2 vector were injected subcutaneously into the flank of NIH-III nude mice (*NIH-Lyst ^bg^Foxn1^nu^Btk^xid^*) (Charles River Laboratory, NY) (5 mice per group). The animals were maintained in a pathogen-free barrier facility at the University of Illinois at Chicago Biological Resources Laboratory, and closely monitored by animal facility staff. Four weeks after injection, tumors were isolated and the weight (gm) and volume (mm^3^) of tumors were determined. Total RNA was isolated from the tumor, quantitative real-time RT-PCR were performed, as described in our recent report [10], to validate SBP1 constitutive expression in the xenografts. All procedures were conducted according to the Animal Care and Use guideline and were approved by the University of Illinois at Chicago Animal Care Committee.

## Results and Discussion

### SBP1 Promoter Was Highly Methylated in Human Colon Cancer Tissues

**Figure 1:**
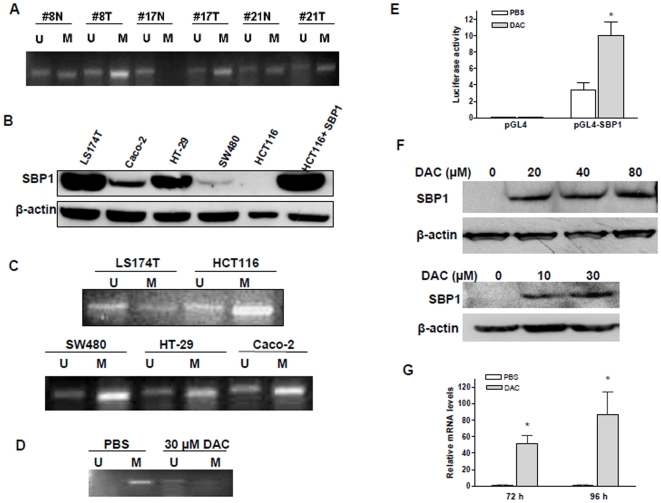
SBP1 promoter was hypermethylated in human cancer tissues and colon cancer cell lines, which silenced gene expression and was revered by 5-Aza-2′-Deoxycytidine (DAC). A, SBP1 promoter was highly methylated in human colon cancer tissues. Genomic DNA from adjacent normal (N) and adenocarcinoma (T) tissue was isolated, converted and amplified for human SBP1 promoter using MS-PCR. # indicated different patients. Unmethylated (U) and methylated (M) bands were detected by DNA separation on 2% agarose gels with ethidium bromide. B, Western blotting analysis showed various expression of SBP1 in human colon cancer cell lines; HCT116 cells with stable transfection of SBP1 overexpressing plasmid was used as positive control. C, SBP1 promoter was hypermethylated in HCT116 cells. Genomic DNA from LS174T, HCT116, SW480, Caco-2 and HT-29 colon cancer cells was isolated, converted and amplified via MS-PCR for human SBP1 promoter followed by gel separation of total DNA on 2% agarose gels (U, unmethylated DNA; M, methylated DNA). D, DAC reversed SBP1 promoter methylation in HCT116 cells. DNA from HCT116 cells treated with 30 µM of DAC for 96 h was isolated and separated on 2% agarose gels after conversion and MS-PCR. E, Luciferase assay showed that DAC increased SBP1 promoter luciferase activity of HCT116 cells with transfection of a vector containing the full length promoter of SBP1 and with a treatment of 30 µM DAC or PBS for 72 hours. pGL4 vector and Renilla was co-transfected as controls (* p<0.01, compared to PBS). F, DAC restored SBP1 protein expression in HCT116 cells with DAC treatment for 72 h, analyzed by Western blotting. G. SBP1 mRNA was restored by DAC in HCT116 cells (* p<0.01, compared to PBS). Each experiment was performed at least 3 times.

We recently reported that SBP1 protein and mRNA expression was dramatically reduced in human colorectal cancer [10], as well as in other types of cancers. To elucidate the reason of gene expression reduction, we isolated targeted human colon cancer cells and matched normal colonic epithelial cells from colorectal cancer patients with low tumor-SBP1 protein and mRNA expression [10] for SBP1 promoter methylation analysis. Surprisingly, we found indeed that the SBP1 promoter was higher methylated in these tumors compared to their adjacent normal mucosa (Fig. 1A). Although higher sample volumes are needed to signify these results, these data clearly demonstrate that SBP1 promoter methylation may be one of the mechanisms responsible for SBP1 downregulation in human colon cancers.

### SBP1 Promoter Was Hypermethylated in Human Colon Cancer Cells

To validate SBP1 promoter methylation and the SBP1 gene expression, human colon cancer cell lines were employed. First, we found various SBP1 protein levels in several cell lines analyzed by Western blotting (Fig. 1B). We then initially selected two cell lines for methylation analysis: LST174T cells which have highest level of SBP1 and HCT116 cells which have an undetectable SBP1 protein expression. As shown in figure 1C, SBP1 promoter was highly methylated in HCT116 cells, while it was mostly unmethylated in LS174T cells. Subsequent sequencing of excised methylated and unmethylated bands confirmed accuracy of the methylation pattern seen in these two cell lines and did not reveal any obvious mutations in the promoter region of SBP1. We also examined SBP1 promoter methylation status in SW480, Caco-2 and HT-29 cells, which have low, moderate and high expression levels of SBP1 protein, respectively (Fig. 1B). Consistent with SBP1 protein expression, the SBP1 promoter was highly methylated in SW480 cells, moderately methylated in Caco-2 cells and methylated to a lesser extent in HT-29 cells (Fig. 1C). We next treated HCT116 cells with the general demethylating agent 5′-Aza-2′-Deoxycytidine to determine whether the hypermethylated SBP1 promoter could be demethylated. We found that treatment of HCT116 cells with 30 µM of DAC for 96 h decreased SBP1 promoter methylation and increased SBP1 promoter unmethylation, compared to the PBS treatment control (Fig. 1D). This suggests that the SBP1 promoter is regulated through methylation of the proximal CpG island and that promoter hypermethylation silences SBP1 expression in HCT116 human colon cancer cells.

### SBP1 Promoter Demethylation Increased SBP1 Promoter Activity and Rescued SBP1 Expression

Our data demonstrate that the SBP1 promoter is hypermethylated and that the demethylating agent DAC could reverse this case. It is therefore important to elucidate whether promoter demethylation could subsequently increase SBP1 promoter activity and SBP1 mRNA and protein expression. First, we transfected HCT116 cells with a luciferase plasmid containing the full length SBP1 promoter region (pGL4-SBP1) and treated the cells with 30 µM DAC for 72 h to test if DAC treatment increases promoter activity, and found that DAC indeed increased SBP1 promoter activity by 3 fold (Fig. 1E). Consistently, HCT116 cells treated with different concentrations of DAC showed an increased SBP1 protein expression in a dose-dependent manner (Fig. 1F). Additionally, DAC treatment increased SBP1 mRNA levels by 50 folds for 72 h treatment and 89 folds for 96 h treatment in HCT116 cells (Fig. 1G). Although other mechanisms for the regulation of SBP1 can not be ruled out, these experiments suggest that SBP1 expression in human colon cancer cells is silenced in part by its promoter methylation and that SBP1 expression can be rescued by demethylating the promoter region.

### SBP1 Inhibits Cell Proliferation, Increases Apoptosis and Inhibits Cell Migration

**Figure 2:**
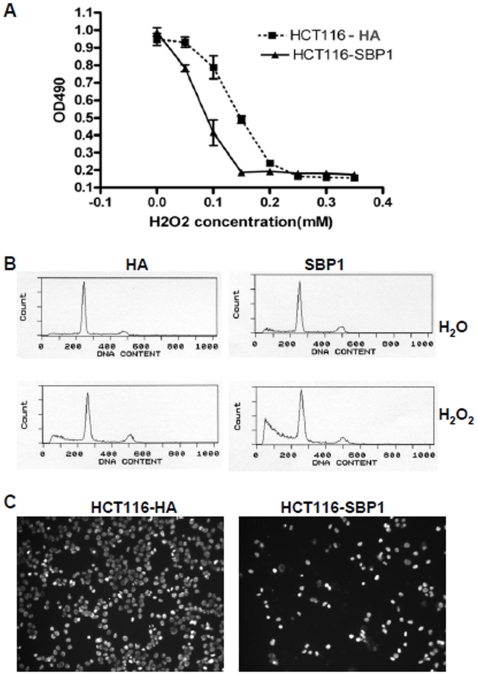
SBP1 had tumor suppressive functions. A, HCT116 cells transfected with HA-SBP1 had an increased sensitivity to H2O2 by MTS proliferation assay compared to the empty vector (HA). The cells were treated with H2O2 for 24 hours. B, HCT116 cells transfected with HA-SBP1 increased H2O2-induced apoptosis by Flow cytometry analysis. Cells were treated with 0.3 mM of H_2_O_2_ for 24 hours. Boxes indicate apoptotic cells (sub-G1). C, SBP1 inhibited cancer cell migration: cell migration of SBP1 overexpressing HCT116 cells and HA-control cells was assessed via transwell chambers. Migrated cells were detected with DAPI staining.

SBP1 has been shown to be involved in the intracellular transport of selenium [2] and to serve as a marker in colonic cell differentiation [10]. SBP1 has also been shown to be decreased in different human cancers. However, its functions in cancers have not yet been defined. Therefore, we wanted to identify the functions SBP1 might have in human colon cancer cells. One hallmark of cancer is uncontrolled cell proliferation. To test if SBP1 might influence cell proliferation, HCT116 cells overexpressing SBP1 were treated with different concentrations of H_2_O_2_ and cell proliferation was analyzed via an MTS assay (Promega, Madison, WI). Although treatment of cells with 0.2 mM H_2_O_2_ itself inhibited cell proliferation in HCT116 cells, it can be appreciated that SBP1 overexpression sensitized HCT116 cells to H_2_O_2_-induced growth inhibition at lower concentrations (<0.2 mM), indicating that SBP1 might play a role in cell cycle regulation (Fig. 2A). FACS analysis revealed that SBP1 overexpression led to an increase of apoptotic cell death (sub-G1 fraction) when cells were treated with H_2_O_2_ (Fig. 2B). Moreover, overexpression of SBP1 in HCT116 cells inhibited human colon cancer cell migration (Fig. 2C). These experiments suggest that SBP1 may have some tumor suppressive functions in human colon cancer cells.

### SBP1 Attenuated Colorectal Cancer Cell Growth in NIH-III Nude Mice

**Figure 3:**
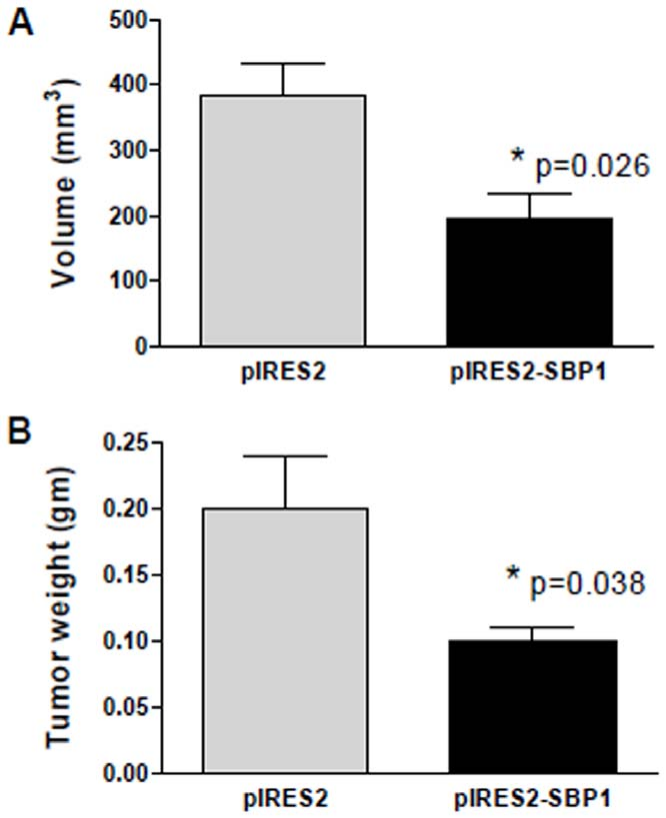
BP1 attenuated colorectal cancer cell growth in NIH-III nude mice. NIH-III nude mice were injected with HCT116 cells stably expressing SBP1 (pIRES2-SBP1) or vector (pIRES2) control. Four weeks after injection, tumor volume (A) and tumor weight (B) was determined.

To test if SBP1 has anti-tumor activities *in vivo*, we performed xenograft experiments in NIH-III nude mice using HCT116 cells which either stably overexpress SBP1 (pIRES2-SBP1) or a control vector (pIRES2). Four weeks after cancer cell injection, tumors were isolated and tumor volume and weight was determined. SBP1 expression in explanted tumors derived from SBP1 overexpressing HCT116 cells was confirmed via RT-PCR showing 607-fold increase at mRNA level. Consistent with *in vitro* data, mice injected with SBP1 overexpressing HCT116 cells had a significantly smaller tumor volume (Fig. 3A) and tumor weight (Fig. 3B) than the mice injected with control cells, indicating that SBP1 has tumor suppressive functions *in vivo* as well.

Taken above, we provide the first evidence that SBP1 promoter is hypermethylated in human colorectal cancer, and that demethylation via DAC increases SBP1 promoter activity as well as rescuing mRNA and protein expression (Fig. 1). Gene silencing is a common mechanism found in many human cancers to inhibit tumor suppressor expression. Our data show that the SBP1 promoter is highly methylated in tumor tissues of colorectal cancer patients, indicating that SBP1 expression in colon cancer is in part controlled through gene silencing. It is critical to investigate whether SBP1 gene silencing is a common mechanism to reduce SBP1 expression in other types of cancer in which SBP1 has been shown to be downregulated. Moreover, due to decreased expression of SBP1 in human cancers, SBP1 has been suggested to have tumor suppressive activities. We believe that we are the first ones to provide evidence that this may indeed be true, in terms of that overexpression of SBP1 in HCT116 cells suppressed cell proliferation, increased apoptotic cell death and decreased cell migration *in vitro* (Fig. 2), and inhibited tumor growth *in vivo* (Fig. 3). However, the exact mechanisms of SBP1 regulation and anti-cancer action remain unknown and need further investigation. In fact, it is currently being investigated in our laboratory. Understanding the regulation and mechanisms of SBP1 tumor suppressor functions will have a great impact in revealing the molecular mechanism of carcinogenesis and in developing more effective chemoprevention and chemotherapies for human malignant diseases.

## References

[1] Chang PW, Tsui SK, Liew C, Lee CC, Waye MM (1997). Isolation, characterization, and chromosomal mapping of a novel cDNA clone encoding human selenium binding protein.. J Cell Biochem.

[2] Porat A, Sagiv Y, Elazar Z (2000). A 56-kDa Selenium-binding Protein Participates in Intra-Golgi Protein Transport.. J Biol Chem.

[3] Scortegagna M, Martin RJ, Kladney RD, Neumann RG, Arbeit JM (2009). Hypoxia-Inducible Factor-1{alpha} Suppresses Squamous Carcinogenic Progression and Epithelial-Mesenchymal Transition.. Cancer Research.

[4] Jeong J-Y, Wang Y, Sytkowski AJ (2009). Human selenium binding protein-1 (hSP56) interacts with VDU1 in a selenium-dependent manner.. Biochemical and Biophysical Research Communications.

[5] Yang M, Sytkowski AJ (1998). Differential Expression and Androgen Regulation of the Human Selenium-binding Protein Gene hSP56 in Prostate Cancer Cells.. Cancer Research.

[6] He QY, Cheung YH, Leung SY, Yuen ST, Chu KM (2004). Diverse proteomic alterations in gastric adenocarcinoma.. Proteomics.

[7] Huang KC, Park DC, Ng SK, Lee JY, Ni X (2006). Selenium binding protein 1 in ovarian cancer.. Int J Cancer.

[8] Guoan C, Hong W, Charles TM, Dafydd GT, Tarek GG (2004). Reduced selenium-binding protein 1 expression is associated with poor outcome in lung adenocarcinomas.. The Journal of Pathology.

[9] Kim H, Kang HJ, You KT, Kim SH, Lee KY (2006). Suppression of human selenium-binding protein 1 is a late event in colorectal carcinogenesis and is associated with poor survival.. Proteomics.

[10] Li T, Yang W, Li M, Byun DS, Tong C (2008). Expression of selenium-binding protein 1 characterizes intestinal cell maturation and predicts survival for patients with colorectal cancer.. Mol Nutr Food Res.

[11] Pass HI, Liu Z, Wali A, Bueno R, Land S (2004). Gene Expression Profiles Predict Survival and Progression of Pleural Mesothelioma.. Clinical Cancer Research.

[12] Jones PA, Baylin SB (2002). The fundamental role of epigenetic events in cancer.. Nat Rev Genet.

[13] Laird PW (2003). The power and the promise of DNA methylation markers.. Nat Rev Cancer.

[14] Toyota M, Ahuja N, Ohe-Toyota M, Herman JG, Baylin SB (1999). CpG island methylator phenotype in colorectal cancer.. Proc Natl Acad Sci U S A.

[15] Li LC, Dahiya R (2002). MethPrimer: designing primers for methylation PCRs.. Bioinformatics.

